# Family history of type 2 diabetes and characteristics of children with newly diagnosed type 1 diabetes

**DOI:** 10.1007/s00125-020-05342-x

**Published:** 2020-12-17

**Authors:** Anna Parkkola, Maaret Turtinen, Taina Härkönen, Jorma Ilonen, Mikael Knip, The Finnish Pediatric Diabetes Register, Mikael Knip, Per-Henrik Groop, Jorma Ilonen, Timo Otonkoski, Riitta Veijola, Alar Abram, Henrikka Aito, Ivan Arkhipov, Elina Blanco-Sequeiros, Jonas Bondestam, Markus Granholm, Maarit Haapalehto-Ikonen, Torsten Horn, Hanna Huopio, Joakim Janer, Christian Johansson, Liisa Kalliokoski, Päivi Keskinen, Anne Kinnala, Maarit Korteniemi, Hanne Laakkonen, Jyrki Lähde, Päivi Miettinen, Päivi Nykänen, Erik Popov, Mari Pulkkinen, Maria Salonen, Pia Salonen, Juhani Sankala, Virpi Sidoroff, Anne-Maarit Suomi, Tuula Tiainen

**Affiliations:** 1 Pediatric Research Center, Children’s Hospital, University of Helsinki and Helsinki University Hospital, Helsinki, Finland; 2 Research Program for Clinical and Molecular Metabolism, Faculty of Medicine, University of Helsinki, Helsinki, Finland; 3 Immunogenetics Laboratory, Institute of Biomedicine, University of Turku, Turku, Finland; 4 Department of Clinical Microbiology, Turku University Hospital, Turku, Finland; 5 Center for Child Health Research, Tampere University Hospital, Tampere, Finland; 6 Folkhälsan Research Center, Helsinki, Finland

**Keywords:** Autoantibodies, Children, Family history, HLA, Type 1 diabetes, Type 2 diabetes

## Abstract

**Aims/hypothesis:**

Shared aetiopathogenetic factors have been proposed in type 1 diabetes and type 2 diabetes and both diseases have been shown to cluster in families. Characteristics related to type 2 diabetes have been described in patients with type 1 diabetes with a positive family history of type 2 diabetes. We wanted to characterise the family history of type 2 diabetes and its possible effects on the phenotype and genotype of type 1 diabetes in affected children at diagnosis.

**Methods:**

A total of 4993 children under the age of 15 years with newly diagnosed type 1 diabetes from the Finnish Pediatric Diabetes Register were recruited (56.6% boys, median age of 8.2 years) for a cross-sectional, observational, population-based investigation. The family history of diabetes at diagnosis was determined by a structured questionnaire, and markers of metabolic derangement, autoantibodies and HLA class II genetics at diagnosis were analysed.

**Results:**

Two per cent of the children had an immediate family member and 36% had grandparents with type 2 diabetes. Fathers and grandfathers were affected by type 2 diabetes more often than mothers and grandmothers. The children with a positive family history for type 2 diabetes were older at the diagnosis of type 1 diabetes (*p* < 0.001), had higher BMI-for-age (*p* = 0.01) and more often tested negative for all diabetes-related autoantibodies (*p* = 0.02).

**Conclusions/interpretation:**

Features associated with type 2 diabetes, such as higher body weight, older age at diagnosis and autoantibody negativity, are more frequently already present at the diagnosis of type 1 diabetes in children with a positive family history of type 2 diabetes.

**Graphical abstract:**

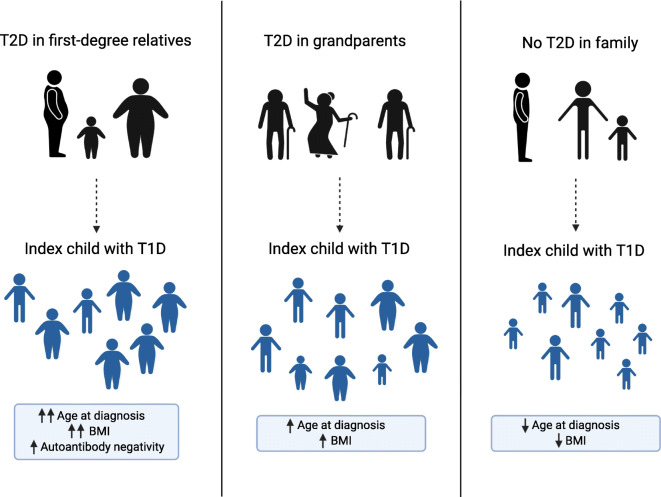

**Supplementary Information:**

The online version contains peer-reviewed but unedited supplementary material available at 10.1007/s00125-020-05342-x.

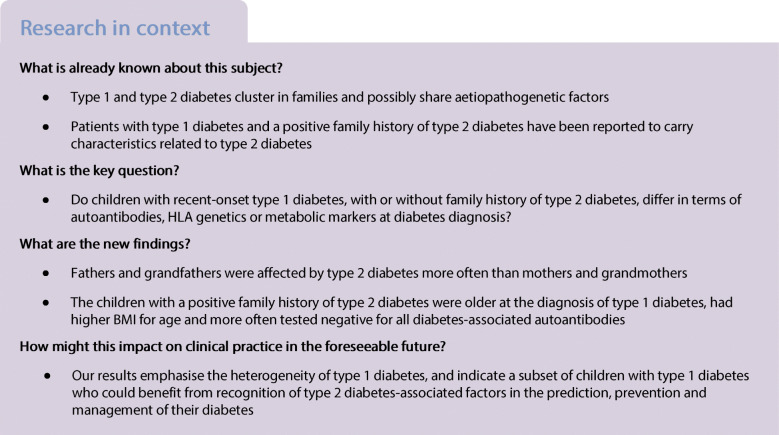



## Introduction

Diabetes mellitus is characterised by hyperglycaemia and inability to control glucose levels. In addition to less frequent subtypes, diabetes has traditionally been classified into two major diseases: type 1 diabetes, which is immune-mediated, and type 2 diabetes, usually related to insulin resistance. This classification is not always straightforward, however, and shared aetiological and pathogenetic processes have been suggested in both major diabetes types [1, 2]. In addition to classical aggressive autoimmunity and insulin resistance pathways, diabetes could develop from the combination of mild autoimmunity and type 2 diabetes-associated risk factors [2].

Epidemiological findings among adult and paediatric populations support the concept of shared aetiopathogenetic factors; type 1 diabetes and type 2 diabetes have been shown to cluster in families in many studies [3–9], although not in all [10–12]. Family history for type 2 diabetes has been reported in 1–70% of patients with type 1 diabetes, depending on the age of the patients and the time elapsed since type 1 diabetes diagnosis, as well as the extent of family members included in the analysis (e.g. first-degree relatives only vs second- and third-degree relatives also included) [3, 5–7, 10–20]. In paediatric patients with type 1 diabetes, 0–13% have been reported to have first-degree relatives and 32–52% extended family members affected by type 2 diabetes [5, 6, 10, 15, 17, 20].

In previous studies, having family members with type 2 diabetes has increased the risk of developing type 1 diabetes [3–8], and is associated, for example, with later onset of type 1 diabetes, higher rate of the metabolic syndrome, a metabolic profile related to insulin resistance in patients with type 1 diabetes (e.g. higher BMI, larger waist circumference, higher triacylglycerols, decreased insulin sensitivity and higher HbA_1c_ concentrations) and greater risk for diabetic complications [15, 16, 18, 21–23]. On the other hand, a positive family history of type 1 diabetes increases the risk to develop type 2 diabetes [24], and is associated with earlier onset of type 2 diabetes, positivity for GAD antibodies (GADA), a more severe insulin deficiency, higher frequency of the HLA-DQB1*03:02/X genotype, which predisposes to type 1 diabetes, and lower frequencies of hypertension and cardiovascular disease, as well as lower BMI and C-peptide levels [25–27].

Accordingly, type 1 diabetes in the presence of a positive family history for type 2 diabetes seems to have many characteristics traditionally associated with type 2 diabetes. Most of the previous studies are from adult populations with long duration of type 1 diabetes, however. In this study of paediatric patients with newly diagnosed type 1 diabetes from the Finnish Pediatric Diabetes Register, we set out to assess whether such characteristics are already present at the time of diagnosis of type 1 diabetes among children. We compared information on demographic characteristics, metabolic status at diagnosis, type 1 diabetes-related autoantibodies and HLA class II genetics among children with or without a family history for type 2 diabetes.

## Methods

### Participants

The data are derived from the population-based Finnish Pediatric Diabetes Register and Sample Repository [28]. The Register invites all children and adolescents diagnosed with diabetes in Finland since 2002 and their family members to participate and covers more than 90% of those diagnosed [29]. Approximately 70% of the participants also provide biological samples for the Repository. Children diagnosed with type 1 diabetes between January 2003 and December 2016 under the age of 15 years with samples available for autoantibody analysis and HLA genotyping were included in this study. The sample collection and characteristics have been described earlier [30]. In brief, 4993 children were included with a male majority (2824/4993, 56.6% boys) and a median age of 8.2 years (ranging from 0.52 to 14.99 years). Children diagnosed under the age of 6 months were excluded, as such infants may have monogenic diabetes. Only one child per family was included as an index case.

Diabetes status and type (type 1, type 2, gestational or other diabetes) of parents, siblings and grandparents were requested using a structured questionnaire [28]. If the family was unsure of the diabetes type, the diabetes doctor or nurse helped with the classification of the disease according to the information provided by the family. For this study, parents or siblings with type 2 diabetes marked in a questionnaire were considered to have type 1 diabetes if two or more autoantibodies were positive, or if monopositivity for GADA was present in conjunction with HLA genotypes predisposing to type 1 diabetes (risk classification of 3–5 [31]). Those with monopositivity for insulin autoantibodies (IAA) or islet cell antibodies (ICA) were not re-classified. Thus, nine parents were re-classified (six fathers, three mothers) as having type 1 diabetes instead of type 2 diabetes. As serum samples for grandparents and 30 parents were not available, such a re-classification was not possible for these relatives and we relied on the self-reported diabetes type. As we were interested in the situation at the time of type 1 diabetes diagnosis of an index child, only relatives with diabetes diagnosed already at this time point were included. Accordingly, 35 relatives with a known diagnosis of type 2 diabetes at a later time point were classified as not having diabetes. The time of diagnosis for 25 relatives was unknown. The autoantibody-negative children with a family member affected by type 2 diabetes were analysed for the coding and promoter sequences with a next-generation sequencing (NGS) panel including *GCK*, *HNF1A*, *HNF4A*, *INS*, *ABCC8*, *KCNJ11* and 38 other genes potentially associated with monogenic diabetes (*AKT2*, *APPL1*, *BLK*, *CEL*, *CISD2*, *DCAF17*, *DNAJC3*, *DYRK1B*, *EIF2AK3*, *FOXP3*, *GATA4*, *GATA6*, *GCK*, *GLIS3*, *HNF1B*, *IER3IP1*, *INSR*, *INSR*, *KLF11*, *LMNA*, *NEUROD1*, *NEUROG3*, *PAX4*, *PCBD1*, *PDX1*, *PIK3R1*, *PLIN1*, *POLD1*, *PPARG*, *PPP1R15B*, *PTF1A*, *RFX6*, *SLC19A2*, *SLC2A2*, *TRMT10A*, *WFS1*, *ZBTB20*, *ZFP57*). None of the children analysed were found to carry any MODY mutation.

The legal guardians of the children and siblings 18 years of age or older gave written, informed consent. Participants aged 10–17 years gave informed assent. The Ethics Committee of the Hospital District of Helsinki and Uusimaa has approved the protocol.

### Autoantibodies

IAA [32], GADA [33], autoantibodies against islet antigen 2 protein (IA-2A) [34] and zinc transporter 8 autoantibodies (ZnT8A) [35] were analysed with specific radiobinding assays. The cut-off limits were 2.80, 5.36, 0.77 and 0.50 relative units (RU), respectively. These limits were determined as the 99th percentiles in more than 350 Finnish non-diabetic children and adolescents. In the 2003 to 2016 Islet Autoantibody Standardization Program, the disease sensitivities and specificities of the assays were 42–62% and 92–99% for IAA, 64–90% and 90–98% for GADA, 62%–72% and 93–100% for IA-2A, and 48–70% and 97–100% for ZnT8A. ICA levels were analysed with indirect immunofluorescence on human group 0 donor pancreas with a detection limit of 2.5 Juvenile Diabetes Foundation (JDF) units [36]. For calculation of the median antibody titres, only samples at or above the cut-off for antibody positivity were included. We excluded samples taken more than 30 days after the diagnosis of type 1 diabetes from the antibody analyses (*n* = 255, 5.1%), since after that the IAA assay also detects antibodies to exogenous insulin.

### HLA typing

We performed HLA typing of major DR-DQ haplotypes as described earlier [37]. HLA class II-conferred risk for type 1 diabetes was estimated by classifying the study participants according to their HLA genotypes into six risk groups ranging from strongly decreased risk (risk group 0) to high risk (risk group 5) [31]. The DRB1*04:01/2/4/5-DQA1*03:01-DQB1*03:02 was denoted as DR4-DQ8, and (DR3)-DQA1*05-DQB1*02 as DR3-DQ2.

### Markers of metabolic decompensation at diagnosis

At diagnosis of type 1 diabetes, blood pH, HbA_1c_, plasma glucose and β-hydroxybutyrate levels of the index children were analysed in local laboratories. Standardised HbA_1c_ values were available only from those diagnosed after the year 2012. We defined ketoacidosis as blood pH <7.30 and severe ketoacidosis as blood pH <7.10. Weight loss, level of consciousness and puberty status by the Tanner scale are determined by a clinician at hospital admission.

### Data handling and statistical analysis

IBM SPSS Statistics 24 (SPSS, IL, USA) and R 3.5.0 package for statistical computing were used for the statistical analyses. For comparing frequencies, cross-tabulation and *χ*^2^ statistics with continuity correction or Fisher’s exact test when appropriate were used. Differences in levels of variables were analysed with one-way ANOVA or Student’s *t* test for parametric, and Kruskal–Wallis test or Mann–Whitney *U* test/Wilcoxon’s rank sum test for non-parametric variables. For variables with more than two groups, linear trend was tested with linear-by-linear test for categorical variables and Jonckheere–Terpsta test for continuous variables. Adjustment for the differences in age and sex was carried out with linear or logistic/ordinal/multinomial regression for parametric or dichotomous/ordinal/categorical variables, and with quantile regression in R (package quantreg 4.54, version 3.5.1) for non-parametric variables [38]. A two-tailed *p* value of 0.05 or less was considered statistically significant. Bonferroni correction for multiple comparisons was not applied due to its overly conservative nature. The z scores for weight-for-age (only for children up to the age of 10 years), height-for-age and BMI-for-age were calculated with WHO AnthroPlus software [39].

## Results

**Fig. 1:**
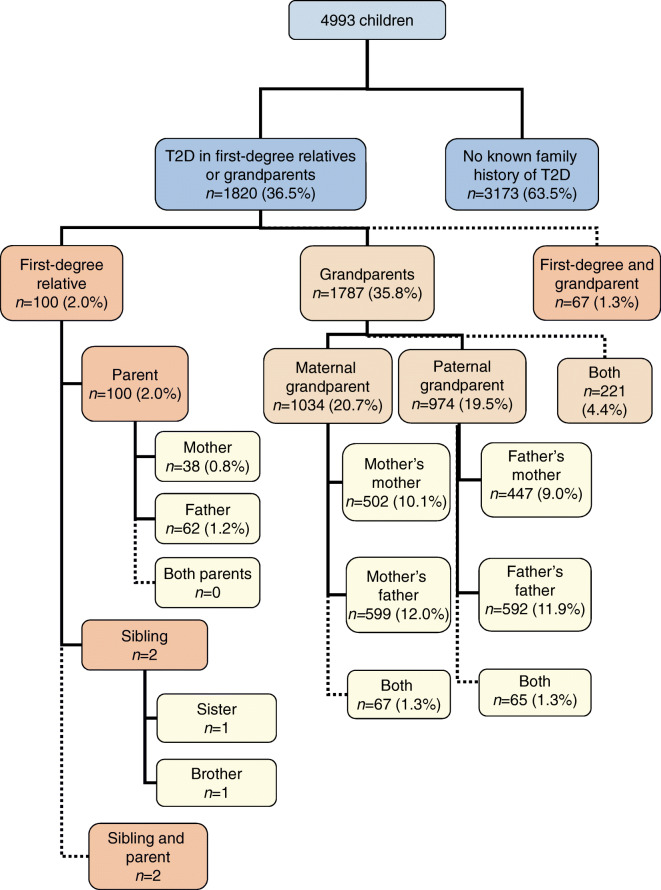
Flowchart displaying the numbers and the proportions of participants with relatives affected by type 2 diabetes in different groups in the total cohort of 4993 children with newly diagnosed type 1 diabetes. The dotted lines indicate those occasions where there are two affected family members, but both family members have already been included in the total number in that category

In total, 100 of 4993 (2%) index children at diagnosis of type 1 diabetes had a mother, a father or a sibling diagnosed with type 2 diabetes. Fathers were more commonly affected than mothers: 1.2% (62/4993) vs 0.8% (38/4993) (*p* = 0.02). Only two children had affected siblings. Both of these were from families where a parent was also affected by type 2 diabetes (Fig. 1). Of the 100 children with first-degree family members with type 2 diabetes, 67 also had grandparents affected by type 2 diabetes.

In contrast, a significant proportion of the children had a grandparent affected by type 2 diabetes: 1787 of 4993 index children (36%) reported at least one affected grandparent. In total, 2140 of the 19,972 grandparents (10.7%) had type 2 diabetes. Again, grandfathers were more commonly affected than grandmothers: 1108 (22.2%) of the index children reported at least one grandfather with type 2 diabetes compared with 896 (17.9%) reporting an affected grandmother (*p* < 0.001). Of the grandfathers, 11.9% (1191/9986), and of the grandmothers, 9.5% (949/9986), reported type 2 diabetes (*p* < 0.001). Grandparents from the maternal side were affected equally often as grandparents from the paternal side of the family (20.7 vs 19.5%, *p* = 0.13).

We compared the children with immediate family members affected by type 2 diabetes (*n* = 100), the children with only grandparents affected by type 2 diabetes (*n* = 1720) and the children with no first-degree relatives or grandparents known to have type 2 diabetes (*n* = 3173) at the time of diagnosis of type 1 diabetes in the index child. The children with a family history of type 2 diabetes were significantly older at diagnosis (Table 1). There was a significant decreasing trend in age at diagnosis: the children with type 2 diabetes in the immediate family members were the oldest, the children with affected grandparents were of intermediate age and the children without a family history of type 2 diabetes were the youngest (*p* for trend <0.001). Accordingly, a similar trend existed for being pubertal, as the older children had more often reached puberty (Tanner stage 2 or higher) (*p* for trend <0.001). Consequently, all the analyses were then adjusted for the differences in age at diagnosis and sex. Adjustment removed the significance in being pubertal. After adjustment, the children with a positive family history of type 2 diabetes had higher BMI-for-age z scores, with the children with affected first-degree relatives being the heaviest and those without family history for type 2 diabetes the lightest (*p* for trend <0.001). After adjustment, there were no differences in the markers of metabolic decompensation at diagnosis, e.g. plasma glucose levels, HbA_1c_, weight loss, frequency of ketoacidosis (Table 2). When comparing autoantibodies, however, the children with first-degree family members with type 2 diabetes were more often negative for all five autoantibodies (Table 3). ICA levels were higher among children with affected grandparents compared with children without a type 2 diabetes family history. There were no significant differences in the frequencies of HLA class II haplotypes or genotypes (electronic supplementary material [ESM] Table 1). However, there was a tendency for more children with a protective HLA genotype and fewer with a high-risk HLA genotype in those with first-degree relatives affected by type 2 diabetes compared with those with affected grandparents or no affected relatives (3.0% vs 0.8% and 0.7% for the protective risk group 0, and 14.0% vs 22.0% and 21.3% for the high-risk group 5).

**Table 1:** Comparison of demographic and anthropometric characteristics among children with first-degree relatives (I), grandparents (II), or no-one in the family (III) affected by type 2 diabetes

Characteristic	n	I. T2D in first-degree relatives (*n* = 100)	II. T2D in grandparents (*n* = 1720)	III. No T2D in family (*n* = 3173)	Unadjusted *p* value^a^	Age- and sex-adjusted *p* value^a^	*p* for trend
Age at diagnosis, years, median (range)	4993	10.6 (1.5–14.7)	8.6 (0.5–14.99)	7.8 (0.5–14.99)	<0.001		<0.001
					I vs II: <0.001		
					I vs III: <0.001		
					II vs III: <0.001		
Sex, male, % (95% CI)	4993	61.0 (51.4, 70.6)	58.1 (55.7, 60.4)	55.6 (53.9, 57.3)	0.16		
Familial T1D (1st degree), % (95% CI)	4993	7.0 (2.0, 12.0)	10.1 (8.7, 11.5)	10.7 (9.6, 11.7)	0.45	0.51	
Pubertal, % (95% CI)	3764	34.9 (23.1, 46.7)	19.5 (17.4, 21.7)	15.4 (13.9, 16.8)	<0.001	0.98	<0.001
Weight-for-age z score, median (range)	3162	0.44 (−1.7 to 6.4)	0.24 (−3.1 to 5.2)	0.17 (−3.5 to 6.1)	0.11	0.23	
Height/length-for-age z score, median (range)	4828	0.64 (−2.1 to 3.5)	0.49 (−2.7 to 4.5)	0.51 (−3.4 to 6.2)	0.78	0.25	
BMI-for-age z score, median (range)	4820	0.28 (−3.4 to 5.5)	−0.21 (−4.9 to 6.0)	−0.27 (−4.6 to 6.0)	0.003	0.01	0.007
						I vs II: 0.01	
						I vs III: 0.02	
						II vs III: 0.03	

^a^The first *p* values refer to comparisons across all groups, whereas the ones below refer to each separate two-way comparison

T1D, type 1 diabetes; T2D, type 2 diabetes

**Table 2:** Comparison of measures of metabolic decompensation at diagnosis of type 1 diabetes among children with first-degree relatives (I), grandparents (II), or no-one in the family (III) affected by type 2 diabetes

Variable	n	I. T2D in first-degree relatives (*n* = 100)	II. T2D in grandparents (*n* = 1720)	III. T2D in family (*n* = 3173)	Unadjusted *p* value	Age- and sex-adjusted *p* value
Plasma glucose, mmol/l, median (range)	4869	22.2 (5.3–71.0)	23.8 (3.5–97.6)	24.0 (3.2–94.6)	0.16	0.62
Ketoacidosis, % (95% CI)	4817	20.6 (12.6, 28.7)	18.6 (16.7, 20.4)	17.5 (16.2, 18.9)	0.54	0.90
Severe ketoacidosis, % (95% CI)	4817	5.2 (0.8, 9.6)	4.4 (3.4, 5.4)	4.7 (4.0, 5.5)	0.81	0.74
pH, median (range)	4817	7.38 (6.82–7.53)	7.38 (6.72–7.57)	7.38 (6.79–7.54)	0.45	0.90
ß-hydroxybutyrate, mmol/l, median (range)	4384	1.7 (0.06–16.7)	1.8 (0–23.5)	1.7 (0–27.0)	0.95	0.76
Impaired consciousness, % (95% CI)	4784	6.1 (1.4, 10.8)	5.6 (4.5, 6.7)	5.3 (4.5, 6.1)	0.91	0.94
HbA_1c_, mmol/mol, mean (SD)	841	101.9 (33.1)	93.8 (28.1)	93.2 (27.3)	0.27	0.67
HbA_1c_, %, mean (SD)	841	11.5 (3.0)	10.7 (2.6)	10.7 (2.5)	0.27	0.68
Weight loss, %, median (range)	4610	5.2 (0–24)	5.5 (0–35)	5.1 (0–40)	0.26	0.25
Duration of symptoms, %	4614				0.03	0.18
No symptoms		0	1.1	0.9		
<1 week		22.7	22.7	22.5		
1–4 weeks		47.4	55.9	58.7		
>4 weeks		29.9	20.4	17.9		

T2D, type 2 diabetes

**Table 3:** Comparison of type 1 diabetes-related autoantibodies among children with first-degree relatives (I), grandparents (II), or no-one in the family (III) affected by type 2 diabetes

Variable	n	I. T2D in first-degree relatives (*n* = 100)	II. T2D in grandparents (*n* = 1720)	III. No T2D in family (*n* = 3173)	Unadjusted *p* value	Age- and sex-adjusted *p* value^a^
Autoantibodies						
ICA, % (95% CI)	4738	85.1 (77.9, 92.3)	91.9 (90.6, 93.3)	91.9 (90.9, 92.8)	0.06	0.16
ICA, JDFU, median (range)	4347	56.5 (3–4096)	64.0 (3–4096)	49.0 (3–5120)	0.02	0.002
						II vs III: <0.002
IAA, % (95% CI)	4738	26.6 (17.7, 35.5)	42.7 (40.3, 45.1)	43.7 (41.9, 45.5)	0.004	0.16
IAA, RU, median (range)	2037	10.5 (3.2–317.0)	10.1 (2.9–7809)	10.3 (2.8–484.9)	0.63	0.91
IA-2A, % (95% CI)	4738	68.1 (58.7, 77.5)	75.6 (73.5, 77.6)	75.0 (73.5, 76.5)	0.27	0.22
IA-2A, RU, median (range)	3556	96.0 (1.0–225.6)	105.4 (0.8–501.0)	105.9 (0.8–553.3)	0.53	0.76
GADA, % (95% CI)	4738	64.9 (55.2, 74.5)	68.3 (66.0, 70.5)	65.4 (63.7, 67.1)	0.13	0.12
GADA, RU, median (range)	3144	34.3 (5.8–319.5)	35.7 (5.4–15,839)	36.1 (5.4–24,849.0)	0.87	0.93
ZnT8A, % (95% CI)	4738	73.4 (64.5, 82.3)	71.3 (69.1, 73.5)	68.3 (66.6, 69.9)	0.08	0.20
ZnT8A, RU, median (range)	3289	10.5 (0.6–170.7)	12.8 (0.5–209.3)	11.8 (0.5–1201.9)	0.88	0.80
Positive antibody responses, median (mean)	4738	3 (3.2)	4 (3.5)	4 (3.4)	0.10	0.35
Antibody multipositive, % (95% CI)	4738	85.1 (77.9, 92.3)	93.0 (91.8, 94.3)	92.4 (91.5, 93.4)	0.02	0.05
Antibody negative, % (95% CI)	4738	7.4 (2.1, 12.8)	2.0 (1.3, 2.7)	2.3 (1.7, 2.8)	0.01	0.02
						I vs II: <0.02
						I vs III: 0.02

^a^The first *p* values refer to comparisons across all groups, while the ones below refer to each separate two-way comparison

‘Multipositive’ means positive for at least two of the measured autoantibodies

JDFU, Juvenile Diabetes Foundation units; T1D, type 1 diabetes; T2D, type 2 diabetes

When the children with affected first-degree relatives or grandparents were pooled together, those with a positive family history for type 2 diabetes (*n* = 1820) were older, and after age and sex adjustment had higher BMI-for-age z scores and higher levels of ICA compared with children without a type 2 diabetes family history (ESM Table 2). There were no differences in HLA genetics.

## Discussion

In our cohort of children newly diagnosed with type 1 diabetes, only 2% of the children reported type 2 diabetes patients among their immediate family members, whereas type 2 diabetes in grandparents was common (36% of the children reported affected grandparents). These figures are in line with those previously reported for children newly diagnosed with type 1 diabetes: in Sweden, 1.7% reported affected immediate family members and 31% reported affected grandparents [5], and in Serbia the figures were 13% and 34%, respectively [6]. In our study, type 2 diabetes seemed more prevalent among fathers and grandfathers compared with mothers and grandmothers. Previous reports from the time of type 1 diabetes diagnosis agree with this result [3, 6, 20], whereas surveys on children [10] and adults [40] with longer duration of type 1 diabetes report equal male–female distribution or even higher maternal prevalence of type 2 diabetes [16]. Our result concurs with epidemiological findings of type 2 diabetes being in general more common among men, and of men being diagnosed with type 2 diabetes at a younger age than women [41]. For reports with maternal overrepresentation, recollection and ascertainment biases in the form of mothers possibly being more aware of the diseases of their relatives and being the parent responsible for answering the study questions might play a role.

We noted an older age at diagnosis of type 1 diabetes among children with a family history for type 2 diabetes. A possible source of bias in this cross-sectional setting is that older patients have in general older family members who are, by definition, more likely to have developed type 2 diabetes. However, our results are in accordance with many previous findings in adults and children [5, 15, 19, 42]. Although, in a previous study from Finland, older age at onset of type 1 diabetes was observed only in adults with a positive family history of type 2 diabetes, and not among those under the age of 15 years [16].

In age- and sex-adjusted analyses, the children with a family history for type 2 diabetes were heavier according to the BMI-for-age z score. This is in accordance with previous reports in children and adults [15, 16], although not all studies agree [19, 22]. The finding is easily explained, as obesity is an important risk factor for type 2 diabetes, and obesity clusters in families due to genetic and lifestyle-based reasons. Accordingly, it seems that the family history of type 2 diabetes translates to higher BMI already at the diagnosis of childhood type 1 diabetes.

A novel finding is that children with newly diagnosed type 1 diabetes were more likely to test negative for diabetes-related autoantibodies at diagnosis if they had family members with type 2 diabetes. In the Diabetes Control and Complications Trial study among adults with longstanding type 1 diabetes, no association was found with autoantibodies and family history of type 2 diabetes [23]. However, only GADA and IA-2A were analysed in that study. Higher frequency of autoantibody-negative patients resonates well with the previous findings of type 2 diabetes-related characteristics among type 1 diabetes patients with a positive family history for type 2 diabetes. Among patients with longer duration of type 1 diabetes, markers related to the metabolic syndrome and insulin resistance have been associated with a positive family history for type 2 diabetes [15, 16, 18, 21–23]. Additionally, patients with newly diagnosed type 1 diabetes have been observed to enter clinical remission more often if they have relatives with type 2 diabetes compared with those without type 2 diabetes in the family [43]. Such patients might have factors related to insulin resistance contributing to the development of diabetes and leading to lower need for aggressive autoimmunity for the disease development. This hypothesis is supported by the distinct features discovered among patients who have developed type 1 diabetes despite having no or only one autoantibody. Such patients often carry the type 2 diabetes-associated *TCF7L2* gene variant [44] and are more often overweight compared with patients with a more severe autoimmune reaction, i.e. multiple autoantibodies [45]. In summary, the results reported by us and by others offer accumulating evidence that in a subset of children with type 1 diabetes, pathogenetic factors related to type 2 diabetes might be involved in the disease development. This may suggest milder autoimmunity in the pathogenesis of type 1 diabetes in these patients with characteristics of both type 1 and 2 diabetes. In such patients, the glycaemic control homeostasis could already be compromised at baseline due to genetic and lifestyle-related factors, and even a mild autoimmune reaction could lead to insufficient insulin action and development of clinical diabetes. Importantly, despite suggesting less aggressive autoimmunity, we detected no indication for a milder metabolic disturbance (e.g. lower plasma glucose levels or less ketoacidosis) at the diagnosis of type 1 diabetes among those with a family history for type 2 diabetes. This would mean that despite the milder autoimmune reaction, this subset of children are not protected from the life-threatening glycaemic crisis at diagnosis and require as rapid medical intervention as the children with a classical severe autoimmune reaction. Larger studies are needed to confirm whether this is the case.

In accordance with previous studies [16, 19], we detected no significant differences in HLA genetics between children with and children without a family history for type 2 diabetes. A tendency existed, however, for more children with protective HLA genetics in the group with first-degree relatives affected by type 2 diabetes. It is likely that this group could have genetic factors associated with type 2 diabetes, but unfortunately these were not analysed in our cohort.

Our study is a cross-sectional, observational investigation of a large, population-based sample of children with newly diagnosed type 1 diabetes. We wanted to investigate the effects of a positive family history for type 2 diabetes on metabolic characteristics, autoantibodies and HLA genetics in children with newly diagnosed type 1 diabetes, as information among paediatric patients at diagnosis is lacking. To our knowledge, the current survey is the first study to compare markers of metabolic derangement and autoantibodies at diagnosis of type 1 diabetes according to family history of type 2 diabetes. Our study is based on a large, population-based register and we have samples available for autoantibody and HLA analyses from a large sample. Consequently, our data are limited to the time of diagnosis of type 1 diabetes of the index children, and we do not have follow-up data available to determine possible later development of type 2 diabetes in family members. Studies with follow-up data might result in a higher number of children with a positive type 2 diabetes family history and thus stronger power for the comparisons. Additionally, our study does not include a control group and thus we are unable to compare the prevalence of type 2 diabetes among family members of type 1 diabetes patients and of control participants. As we do not have measurements such as BMI or weight to help in the classification of diabetes in the relatives, we rely mostly on self-reports of the families. Furthermore, family members of a patient with one type of diabetes are de facto at increased risk for the same type of diabetes, which complicates classification in clinical and research settings. To be better able to analyse the possible pathogenetic factors behind the different subsets of patients, factors related to the metabolic syndrome, for example, lipid levels or measures of insulin resistance from the index children, would have been interesting. Unfortunately, such measurements were not possible in such a large cohort.

If a child with diabetes tests negative for disease-associated autoantibodies at diagnosis, one has to consider alternative diagnoses, such as type 2 diabetes or monogenic diabetes. In follow-up studies of risk individuals we have seen that sometimes a child that tests autoantibody-positive preclinically is autoantibody-negative at diagnosis. We can not totally exclude the possibility that some of the autoantibody-negative children in our study cohort had in fact type 2 diabetes or some other diabetes type instead of type 1 diabetes. We believe this number to be small, however, as type 2 diabetes in children in Finland is still rare, and careful diagnostic procedures are performed in paediatric units in Finland. Additionally, to exclude any cases of monogenic diabetes, children diagnosed with type 1 diabetes at a very young age were excluded and autoantibody-negative children with a first-degree relative affected by type 2 diabetes underwent NGS analysis of 44 genes potentially associated with monogenic diabetes, including seven MODY genes (*GCK*, *HNF1A*, *HNF1B*, *HNF4A*, *INS*, *ABCC8*, *KCNJ11*). There could also be the rare possibility that a child might have autoantibodies to a so far uncharacterised antigen.

In conclusion, our findings emphasise the heterogeneity of diabetes and imply possible shared aetiopathogenetic factors with type 2 diabetes in some children with type 1 diabetes. Further studies are needed to better understand this heterogeneity in the pathogenetic processes. Nevertheless, children with a positive family history for type 2 diabetes could benefit from recognition of type 2-related risk factors in the prediction, prevention and management of their diabetes.

## Supplementary Information

ESM (PDF 156 kb)

## Data Availability

Data are available on reasonable request from the corresponding author.

## Abbreviations

GADA: GAD antibodies
IAA: Insulin autoantibodies
IA-2A: Autoantibodies against islet antigen 2 protein
ICA: Islet cell antibodies
NGS: Next-generation sequencing
RU: Relative units
ZnT8A: Zinc transporter 8 autoantibodies
