# Supplementary material for: Family history of type 2 diabetes and characteristics of children with newly diagnosed type 1 diabetes
Source: Diabetologia. 2020 Dec 17;64(3):581–90. doi: 10.1007/s00125-020-05342-x (PMC7864815; doi:10.1007/s00125-020-05342-x)
Supplement: Supplementary file 1 — (PDF 156 kb) [file 125_2020_5342_MOESM1_ESM.pdf]

Electronic Supplementary Material (ESM)

This appendix has been provided by the authors to give readers additional information about their work

Supplement to: Parkkola A, Turtinen M, Härkönen T, Ilonen J, Knip M, and the Finnish Pediatric Diabetes Register. Family history of type 2 diabetes and characteristics of children with newly diagnosed type 1 diabetes. *Diabetologia*.

-Supplemental Table 1
-Supplemental Table 2
-Investigators of the Finnish Pediatric Diabetes Register

ESM Table 1 Comparison of HLA class II haplotypes and genotypes among 4,993 children with first-degree relatives (I), grandparents (II), or none in the family (III) affected by type 2 diabetes (T2D). 95% confidence intervals (CI) are shown.

	I. T2D in first-degree relatives (n=100)	II. T2D in grandparents (n=1,720)	III. No T2D in family (n=3,173)	Unadjusted *p* value	Age and sex adjusted *p* value
**HLA class II genetics**					
DR3-DQ2/DR4-DQ8, % (95%CI)	13.0 (6.4-19.6)	22.0 (20.0-23.9)	21.2 (19.8-22.7)	0.10	0.14
DR3-DQ2/x*, % (95%CI)	18.0 (10.5-25.5)	14.7 (13.0-16.3)	15.6 (14.3-16.9)	0.51	0.50
DR4-DQ8/y†, % (95%CI)	52.0 (42.2-61.8)	48.7 (46.3-51.0)	47.4 (45.7-49.2)	0.51	0.53
x*/y†, % (95%CI)	17.0 (9.6-24.4)	14.7 (13.0-16.4)	15.7 (14.5-17.0)	0.58	0.51
DR3-DQ2 positive, % (95%CI)	31.0 (21.9-40.1)	36.6 (34.4-38.9)	36.8 (35.2-38.5)	0.49	0.60
DR4-DQ8 positive, % (95%CI)	65.0 (55.7-74.3)	70.6 (68.5-72.8)	68.6 (67.0-70.3)	0.23	0.20
DR3-DQ2 homozygote, % (95%CI)	4.0 (0.2-7.8)	3.0 (2.2-3.8)	3.0 (2.4-3.6)	0.75	0.88
DR4-DQ8 homozygote, % (95%CI)	10.0 (4.1-15.9)	7.6 (6.3-8.8)	8.4 (7.4-9.3)	0.47	0.47
Risk group, %				0.35	0.60
0	3.0	0.8	0.7		
1	3.0	1.9	2.1		
2	15.0	15.6	16.1		
3	27.0	22.3	22.8		
4	38.0	37.4	36.9		
5	14.0	22.0	21.3		

* x ≠ DR4-DQ8
†y ≠ DR3-DQ2

ESM Table 2 Comparison of demographic characteristics, metabolic status, autoantibodies, and HLA class II genetics between children with first-degree relatives or grandparents (I) with type 2 diabetes (T2D) and children with no type 2 diabetes in the family (II). 95% confidence intervals (CI) shown.

	n	I. T2D in first-degree relatives or grandparents (n=1,820)	II. No T2D in family (n=3,173)	Unadjusted *p* value	Age and sex adjusted *p* value
**Demographics**					
Age at diagnosis, yr, median (range)	4,993	8.7 (0.5-14.99)	7.8 (0.5-14.99)	**<0.001**	
Sex, male, % (95%CI)	4,993	58.2 (56.0-60.5)	55.6 (53.9-57.3)	0.07	
Familial T1D (1.st degree), % (95%CI)	4,993	9.9 (8.6-11.3)	10.7 (9.6-11.7)	0.46	0.49
Pubertal, % (95%CI)	3,764	20.3 (18.1-22.4)	15.4 (13.9-16.8)	**<0.001**	0.99
Weight-for-age, z-score, median (range)	3,162	0.25 (-3.1-6.4)	0.17 (-3.5-6.1)	0.23	0.07
Height/length-for-age, z-score, median (range)	4,828	0.50 (-2.7-4.5)	0.51 (-3.4-6.2)	0.88	0.73
BMI-for-age, z-score, median (range)	4,820	-0.20 (-4.9-6.0)	-0.27 (-4.6-6.0)	**0.01**	**0.01**
**Metabolic decompensation at diagnosis**					
Plasma glucose, mmol/l, median (range)	4,869	23.7 (3.5-97.6)	24.0 (3.2-94.6)	0.60	0.40
Ketoacidosis, % (95%CI)	4,817	18.7 (16.9-20.5)	17.5 (16.2-18.9)	0.34	0.66
Severe ketoacidosis, % (95%CI)	4,817	4.4 (3.5-5.4)	4.7 (4.0-5.5)	0.73	0.44
pH, median (range)	4,817	7.38 (6.72-7.57)	7.38 (6.79-7.54)	0.22	0.71
ß-hydroxybutyrate, mmol/l, median (range)	4,384	1.8 (0-23.5)	1.7 (0-27.0)	0.76	0.97
Impaired consciousness, % (95%CI)	4,784	5.6 (4.5-6.7)	5.3 (4.5-6.1)	0.75	0.76
HbA1c, mmol/mol, mean (SD)	841	94.4 (28.6)	93.2 (27.3)	0.52	0.82
HbA1c, %, mean (SD)	841	10.8 (2.6)	10.7 (2.5)	0.51	0.83
Weight loss, kg, median, (range)	4,671	1.4 (0-30.0)	1.0 (0-23.0)	**0.01**	0.32
Weight loss, %, median (range)	4,610	5.5 (0-35)	5.1 (0-40)	0.10	0.30
Duration of symptoms, %	4,614			0.06	0.24
No symptoms		1.0	0.9		
< 1 week		22.7	22.5		
1-4 weeks		55.4	58.7		
> 4 weeks		20.9	17.9		
**Autoantibodies**					
ICA, % (95%CI)	4,738	91.6 (90.3-92.9)	91.9 (90.9-92.8)	0.76	0.96
ICA, JDFU, median (range)	4,347	64.0 (3-4,096)	49.0 (3-5,120)	**0.008**	**0.001**
IAA, % (95%CI)	4,738	41.8 (39.5-44.1)	43.7 (41.9-45.5)	0.22	0.47
IAA, RU, median (range)	2,037	10.1 (2.9-7,809.0)	10.3 (2.8-484.9)	0.34	0.88
IA-2A, % (95%CI)	4,738	75.1 (73.1-77.2)	75.0 (73.5-76.5)	0.94	0.95
IA-2A, RU, median (range)	3,556	105.1 (0.8-501.0)	105.9 (0.8-553.3)	0.83	0.66
GADA, % (95%CI)	4,738	68.1 (65.9-70.3)	65.4 (63.7-67.1)	0.06	0.06
GADA, RU, median (range)	3,144	35.7 (5.4-15,839.0)	36.1 (5.4-24,849.0)	0.66	0.96

ZnT8A, % (95%CI)	4,738	71.4 (69.3-73.5)	68.3 (66.6-69.9)	**0.03**	0.08
ZnT8A, RU, median (range)	3,289	12.6 (0.5-209.3)	11.8 (0.5-1,201.9)	0.81	0.58
Positive antibody responses, median (mean)	4,738	4 (3.5)	4 (3.4)	0.27	0.56
Antibody negative, % (95%CI)	4,738	2.3	2.3 (1.7-2.8)	0.99	0.78
Antibody multipositive, % (95%CI)	4,738	92.6	92.4 (91.5-93.4)	0.87	0.50
**HLA class II genetics**					
DR3-DQ2/DR4-DQ8, % (95%CI)	4,993	21.5 (19.6-23.4)	21.2 (19.8-22.7)	0.87	0.61
DR3-DQ2/x*, % (95%CI)	4,993	14.8 (13.2-16.5)	15.6 (14.3-16.9)	0.50	0.42
DR4-DQ8/y†, % (95%CI)	4,993	48.8 (46.5-51.1)	47.4 (45.7-49.2)	0.35	0.35
x*/y†, % (95%CI)	4,993	14.8 (13.2-16.5)	15.7 (14.5-17.0)	0.42	0.28
DR3-DQ2 positive, % (95%CI)	4,993	36.3 (34.1-38.5)	36.8 (35.2-38.5)	0.74	0.86
DR4-DQ8 positive, % (95%CI)	4,993	70.3 (68.2-72.4)	68.6 (67.0-70.3)	0.23	0.14
DR3-DQ2 homozygote, % (95%CI)	4,993	3.0 (2.2-3.8)	3.0 (2.4-3.6)	1.00	0.92
DR4-DQ8 homozygote, % (95%CI)	4,993	7.7 (6.5-8.9)	8.4 (7.4-9.3)	0.42	0.34
Risk group, %	4,993			0.95	0.36
0		0.9	0.7		
1		2.0	2.1		
2		15.5	16.1		
3		22.5	22.8		
4		37.5	36.9		
5		21.5	21.3		

* x ≠ DR4-DQ8

†y ≠ DR3-DQ2

The Finnish Pediatric Diabetes Register comprises the following investigators:

***Principal Investigator*:** Mikael Knip (Children's Hospital, Helsinki University Hospital)

Steering Committee: Per-Henrik Groop (Folkhälsan Research Center), Jorma Ilonen (Immunogenetics Laboratory, University of Turku), Timo Otonkoski (Children's Hospital, Helsinki University Hospital), Riitta Veijola (Department of Pediatrics, Oulu University Hospital).

Locally responsible investigators:

Alar Abram (Department of Pediatrics, Kanta-Häme Central Hospital), Henrikka Aito (Department of Pediatrics, HUS Porvoo Hospital), Ivan Arkhipov (Department of Pediatrics, Mehiläinen Länsi-Pohja Central Hospital), Elina Blanco-Sequeiros (Department of Pediatrics, Central Ostrobothnia Central Hospital), Jonas Bondestam (Department of Pediatrics, HUS Lohja Hospital), Markus Granholm (Department of Pediatrics, Jakobstad Hospital), Maarit Haapalehto-Ikonen (Department of Pediatrics, Rauma Hospital), Torsten Horn (Department of Pediatrics, Central Hospital of Central Finland), Hanna Huopio (Department of Pediatrics, Kuopio University Hospital), Joakim Janer (Department of Pediatrics, HUS Raasepori Hospital), Christian Johansson (Department of Pediatrics, Åland Central Hospital), Liisa Kalliokoski (Department of Pediatrics, Kainuu Central Hospital), Päivi Keskinen (Department of Pediatrics, Tampere University Hospital), Anne Kinnala (Department of Pediatrics, Turku University Central Hospital), Maarit Korteniemi (Department of Pediatrics, Central Hospital of Lapland), Hanne Laakkonen (Department of Pediatrics, HUS Hyvinkää Hospital), Jyrki Lähde (Department of Pediatrics, Satakunta Central Hospital), Päivi Miettinen (HUS New Children's Hospital). Päivi Nykänen (Department of Pediatrics, Mikkeli Central Hospital), Erik Popov (Department of Pediatrics, Vaasa Central Hospital), Mari Pulkkinen (Department of Pediatrics, HUS Jorvi Hospital), Maria Salonen (Department of Pediatrics, Kymenlaakso Central Hospital), Pia Salonen (Department of Pediatrics, Päijät-

Häme Central Hospital), Juhani Sankala (Department of Pediatrics, Savonlinna Central Hospital), Virpi Sidoroff (Department of Pediatrics, North Karelia Central Hospital), Anne-Maarit Suomi (Department of Pediatrics, South Ostrobothnia Central Hospital, Tuula Tiainen (Department of Pediatrics, South Karelia Central Hospital), Riitta Veijola (Department of Pediatrics, Oulu University Hospital)
